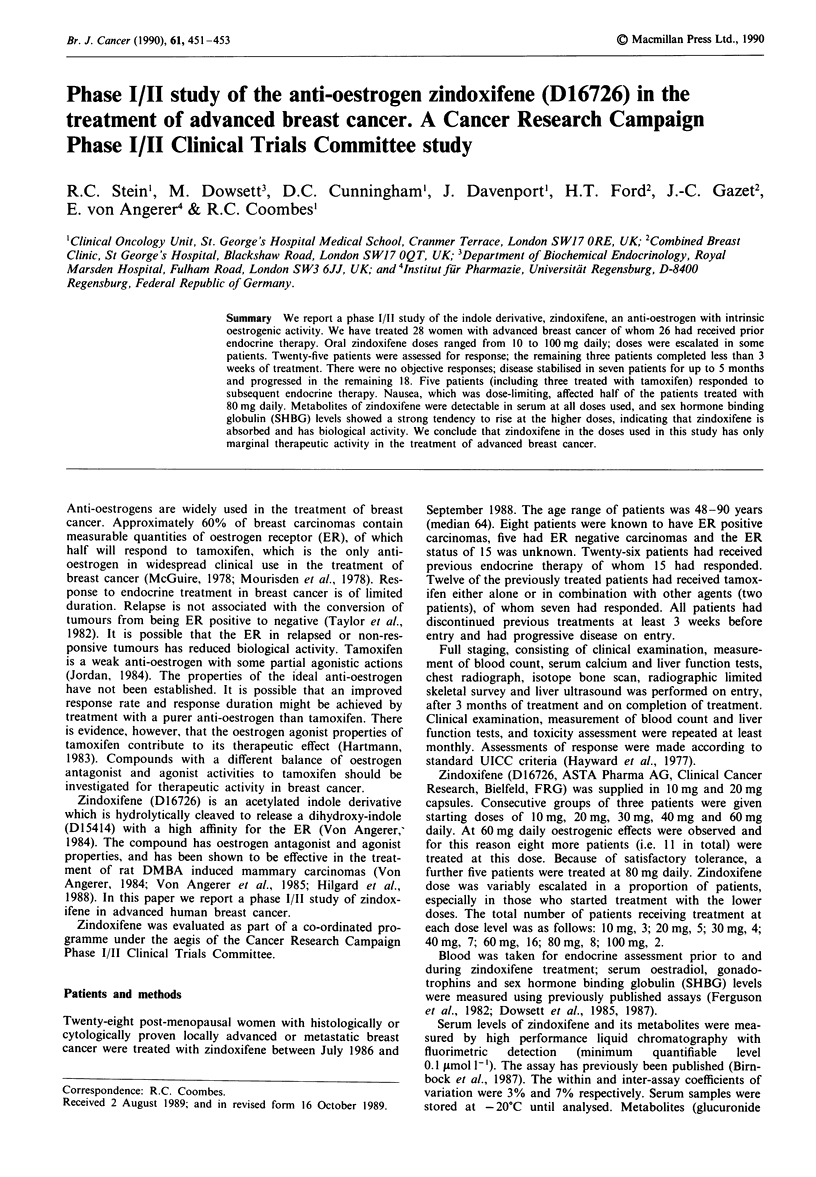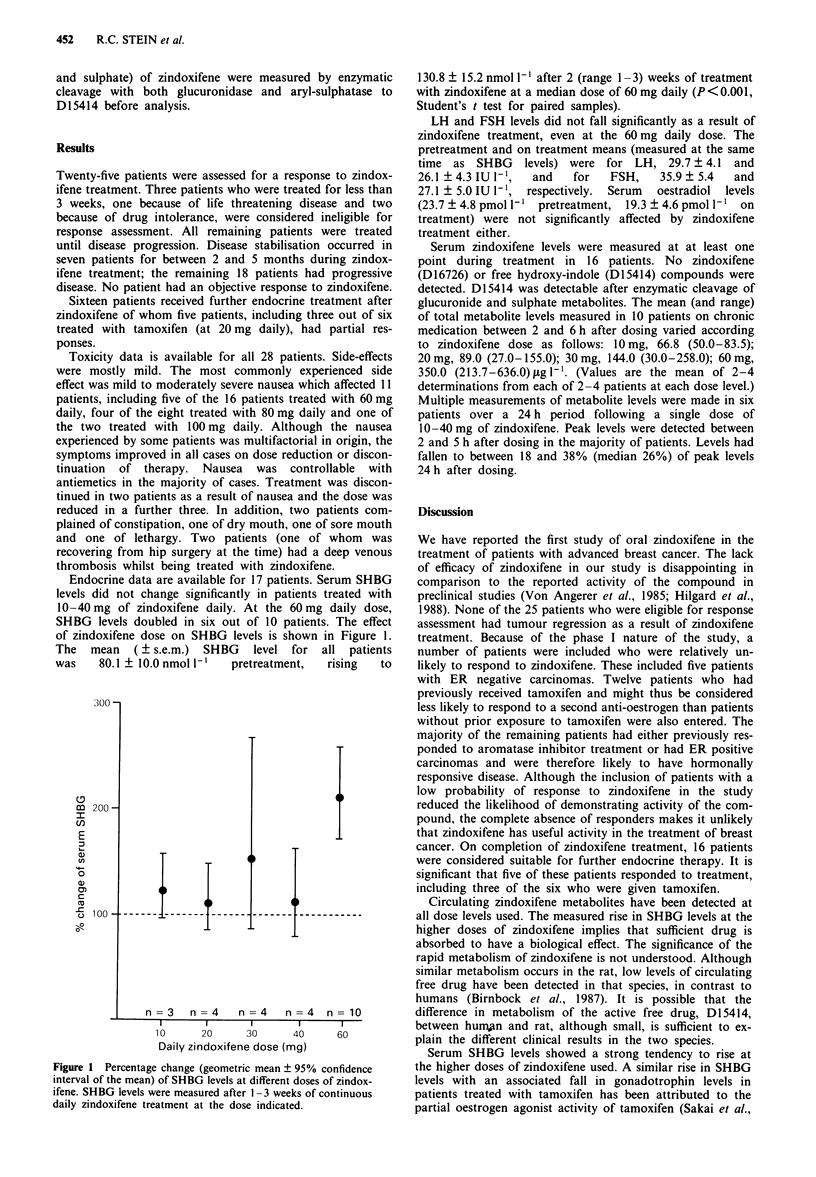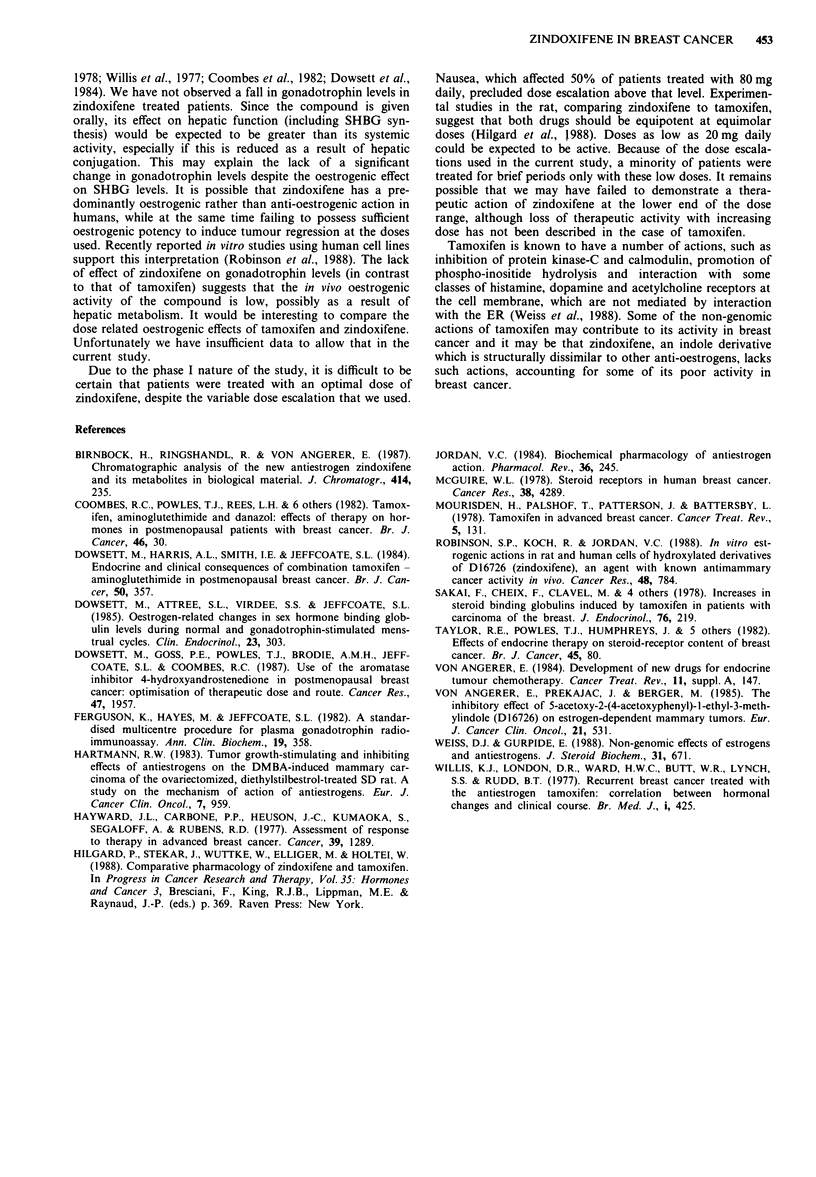# Phase I/II study of the anti-oestrogen zindoxifene (D16726) in the treatment of advanced breast cancer. A Cancer Research Campaign Phase I/II Clinical Trials Committee study.

**DOI:** 10.1038/bjc.1990.99

**Published:** 1990-03

**Authors:** R. C. Stein, M. Dowsett, D. C. Cunningham, J. Davenport, H. T. Ford, J. C. Gazet, E. von Angerer, R. C. Coombes

**Affiliations:** Clinical Oncology Unit, St. George's Hospital Medical School, London, UK.

## Abstract

We report a phase I/II study of the indole derivative, zindoxifene, an anti-oestrogen with intrinsic oestrogenic activity. We have treated 28 women with advanced breast cancer of whom 26 had received prior endocrine therapy. Oral zindoxifene doses ranged from 10 to 100 mg daily; doses were escalated in some patients. Twenty-five patients were assessed for response; the remaining three patients completed less than 3 weeks of treatment. There were no objective responses; disease stabilised in seven patients for up to 5 months and progressed in the remaining 18. Five patients (including three treated with tamoxifen) responded to subsequent endocrine therapy. Nausea, which was dose-limiting, affected half of the patients treated with 80 mg daily. Metabolites of zindoxifene were detectable in serum at all doses used, and sex hormone binding globulin (SHBG) levels showed a strong tendency to rise at the higher doses, indicating that zindoxifene is absorbed and has biological activity. We conclude that zindoxifene in the doses used in this study has only marginal therapeutic activity in the treatment of advanced breast cancer.


*Br. J. Cancer* (1990), **61**, 451–453 © Macmillan Press Ltd., 1990

### Phase I/II study of the anti-oestrogen zindoxifene (D16726) in the treatment of advanced breast cancer. A Cancer Research Campaign Phase I/II Clinical Trials Committee study

R.C. Stein[1], M. Dowsett[3], D.C. Cunningham[1], J. Davenport[1], H.T. Ford[2], J.-C. Gazet[2], E. von Angerer[4] & R.C. Coombes[1]

[1]*Clinical Oncology Unit, St. George's Hospital Medical School, Cranmer Terrace, London SW17 0RE, UK;* [2]*Combined Breast Clinic, St George's Hospital, Blackshaw Road, London SW17 0QT, UK;* [3]*Department of Biochemical Endocrinology, Royal Marsden Hospital, Fulham Road, London SW3 6JJ, UK; and* [4]*Institut für Pharmazie, Universität Regensburg, D-8400 Regensburg, Federal Republic of Germany.*

**Summary** We report a phase I/II study of the indole derivative, zindoxifene, an anti-oestrogen with intrinsic oestrogenic activity. We have treated 28 women with advanced breast cancer of whom 26 had received prior endocrine therapy. Oral zindoxifene doses ranged from 10 to 100 mg daily; doses were escalated in some patients. Twenty-five patients were assessed for response; the remaining three patients completed less than 3 weeks of treatment. There were no objective responses; disease stabilised in seven patients for up to 5 months and progressed in the remaining 18. Five patients (including three treated with tamoxifen) responded to subsequent endocrine therapy. Nausea, which was dose-limiting, affected half of the patients treated with 80 mg daily. Metabolites of zindoxifene were detectable in serum at all doses used, and sex hormone binding globulin (SHBG) levels showed a strong tendency to rise at the higher doses, indicating that zindoxifene is absorbed and has biological activity. We conclude that zindoxifene in the doses used in this study has only marginal therapeutic activity in the treatment of advanced breast cancer.

Anti-oestrogens are widely used in the treatment of breast cancer. Approximately 60% of breast carcinomas contain measurable quantities of oestrogen receptor (ER), of which half will respond to tamoxifen, which is the only anti-oestrogen in widespread clinical use in the treatment of breast cancer (McGuire, 1978; Mourisden *et al.*, 1978). Response to endocrine treatment in breast cancer is of limited duration. Relapse is not associated with the conversion of tumours from being ER positive to negative (Taylor *et al.*, 1982). It is possible that the ER in relapsed or non-responsive tumours has reduced biological activity. Tamoxifen is a weak anti-oestrogen with some partial agonistic actions (Jordan, 1984). The properties of the ideal anti-oestrogen have not been established. It is possible that an improved response rate and response duration might be achieved by treatment with a purer anti-oestrogen than tamoxifen. There is evidence, however, that the oestrogen agonist properties of tamoxifen contribute to its therapeutic effect (Hartmann, 1983). Compounds with a different balance of oestrogen antagonist and agonist activities to tamoxifen should be investigated for therapeutic activity in breast cancer.

Zindoxifene (D16726) is an acetylated indole derivative which is hydrolytically cleaved to release a dihydroxy-indole (D15414) with a high affinity for the ER (Von Angerer, 1984). The compound has oestrogen antagonist and agonist properties, and has been shown to be effective in the treatment of rat DMBA induced mammary carcinomas (Von Angerer, 1984; Von Angerer *et al.*, 1985; Hilgard *et al.*, 1988). In this paper we report a phase I/II study of zindoxifene in advanced human breast cancer.

Zindoxifene was evaluated as part of a co-ordinated programme under the aegis of the Cancer Research Campaign Phase I/II Clinical Trials Committee.

### Patients and methods

Twenty-eight post-menopausal women with histologically or cytologically proven locally advanced or metastatic breast cancer were treated with zindoxifene between July 1986 and September 1988. The age range of patients was 48–90 years (median 64). Eight patients were known to have ER positive carcinomas, five had ER negative carcinomas and the ER status of 15 was unknown. Twenty-six patients had received previous endocrine therapy of whom 15 had responded. Twelve of the previously treated patients had received tamoxifen either alone or in combination with other agents (two patients), of whom seven had responded. All patients had discontinued previous treatments at least 3 weeks before entry and had progressive disease on entry.

Full staging, consisting of clinical examination, measurement of blood count, serum calcium and liver function tests, chest radiograph, isotope bone scan, radiographic limited skeletal survey and liver ultrasound was performed on entry, after 3 months of treatment and on completion of treatment. Clinical examination, measurement of blood count and liver function tests, and toxicity assessment were repeated at least monthly. Assessments of response were made according to standard UICC criteria (Hayward *et al.*, 1977).

Zindoxifene (D16726, ASTA Pharma AG, Clinical Cancer Research, Bielfeld, FRG) was supplied in 10 mg and 20 mg capsules. Consecutive groups of three patients were given starting doses of 10 mg, 20 mg, 30 mg, 40 mg and 60 mg daily. At 60 mg daily oestrogenic effects were observed and for this reason eight more patients (i.e. 11 in total) were treated at this dose. Because of satisfactory tolerance, a further five patients were treated at 80 mg daily. Zindoxifene dose was variably escalated in a proportion of patients, especially in those who started treatment with the lower doses. The total number of patients receiving treatment at each dose level was as follows: 10 mg, 3; 20 mg, 5; 30 mg, 4; 40 mg, 7; 60 mg, 16; 80 mg, 8; 100 mg, 2.

Blood was taken for endocrine assessment prior to and during zindoxifene treatment; serum oestradiol, gonadotrophins and sex hormone binding globulin (SHBG) levels were measured using previously published assays (Ferguson *et al.*, 1982; Dowsett *et al.*, 1985, 1987).

Serum levels of zindoxifene and its metabolites were measured by high performance liquid chromatography with fluorimetric detection (minimum quantifiable level 0.1 $\mu mol\,l^{-1}$). The assay has previously been published (Birnbock *et al.*, 1987). The within and inter-assay coefficients of variation were 3% and 7% respectively. Serum samples were stored at −20°C until analysed. Metabolites (glucuronide

Correspondence: R.C. Coombes.
Received 2 August 1989; and in revised form 16 October 1989.

and sulphate) of zindoxifene were measured by enzymatic cleavage with both glucuronidase and aryl-sulphatase to D15414 before analysis.

### Results

Twenty-five patients were assessed for a response to zindoxifene treatment. Three patients who were treated for less than 3 weeks, one because of life threatening disease and two because of drug intolerance, were considered ineligible for response assessment. All remaining patients were treated until disease progression. Disease stabilisation occurred in seven patients for between 2 and 5 months during zindoxifene treatment; the remaining 18 patients had progressive disease. No patient had an objective response to zindoxifene.

Sixteen patients received further endocrine treatment after zindoxifene of whom five patients, including three out of six treated with tamoxifen (at 20 mg daily), had partial responses.

Toxicity data is available for all 28 patients. Side-effects were mostly mild. The most commonly experienced side effect was mild to moderately severe nausea which affected 11 patients, including five of the 16 patients treated with 60 mg daily, four of the eight treated with 80 mg daily and one of the two treated with 100 mg daily. Although the nausea experienced by some patients was multifactorial in origin, the symptoms improved in all cases on dose reduction or discontinuation of therapy. Nausea was controllable with antiemetics in the majority of cases. Treatment was discontinued in two patients as a result of nausea and the dose was reduced in a further three. In addition, two patients complained of constipation, one of dry mouth, one of sore mouth and one of lethargy. Two patients (one of whom was recovering from hip surgery at the time) had a deep venous thrombosis whilst being treated with zindoxifene.

Endocrine data are available for 17 patients. Serum SHBG levels did not change significantly in patients treated with 10–40 mg of zindoxifene daily. At the 60 mg daily dose, SHBG levels doubled in six out of 10 patients. The effect of zindoxifene dose on SHBG levels is shown in Figure 1. The mean ($\pm$ s.e.m.) SHBG level for all patients was $80.1 \pm 10.0$ nmol $l^{-1}$ pretreatment, rising to

**Figure 1** Percentage change (geometric mean $\pm$ 95% confidence interval of the mean) of SHBG levels at different doses of zindoxifene. SHBG levels were measured after 1–3 weeks of continuous daily zindoxifene treatment at the dose indicated.

$130.8 \pm 15.2$ nmol $l^{-1}$ after 2 (range 1–3) weeks of treatment with zindoxifene at a median dose of 60 mg daily ($P < 0.001$, Student's $t$ test for paired samples).

LH and FSH levels did not fall significantly as a result of zindoxifene treatment, even at the 60 mg daily dose. The pretreatment and on treatment means (measured at the same time as SHBG levels) were for LH, $29.7 \pm 4.1$ and $26.1 \pm 4.3$ IU $l^{-1}$, and for FSH, $35.9 \pm 5.4$ and $27.1 \pm 5.0$ IU $l^{-1}$, respectively. Serum oestradiol levels ($23.7 \pm 4.8$ pmol $l^{-1}$ pretreatment, $19.3 \pm 4.6$ pmol $l^{-1}$ on treatment) were not significantly affected by zindoxifene treatment either.

Serum zindoxifene levels were measured at at least one point during treatment in 16 patients. No zindoxifene (D16726) or free hydroxy-indole (D15414) compounds were detected. D15414 was detectable after enzymatic cleavage of glucuronide and sulphate metabolites. The mean (and range) of total metabolite levels measured in 10 patients on chronic medication between 2 and 6 h after dosing varied according to zindoxifene dose as follows: 10 mg, 66.8 (50.0–83.5); 20 mg, 89.0 (27.0–155.0); 30 mg, 144.0 (30.0–258.0); 60 mg, 350.0 (213.7–636.0) $\mu g\, l^{-1}$. (Values are the mean of 2–4 determinations from each of 2–4 patients at each dose level.) Multiple measurements of metabolite levels were made in six patients over a 24 h period following a single dose of 10–40 mg of zindoxifene. Peak levels were detected between 2 and 5 h after dosing in the majority of patients. Levels had fallen to between 18 and 38% (median 26%) of peak levels 24 h after dosing.

### Discussion

We have reported the first study of oral zindoxifene in the treatment of patients with advanced breast cancer. The lack of efficacy of zindoxifene in our study is disappointing in comparison to the reported activity of the compound in preclinical studies (Von Angerer *et al.*, 1985; Hilgard *et al.*, 1988). None of the 25 patients who were eligible for response assessment had tumour regression as a result of zindoxifene treatment. Because of the phase I nature of the study, a number of patients were included who were relatively unlikely to respond to zindoxifene. These included five patients with ER negative carcinomas. Twelve patients who had previously received tamoxifen and might thus be considered less likely to respond to a second anti-oestrogen than patients without prior exposure to tamoxifen were also entered. The majority of the remaining patients had either previously responded to aromatase inhibitor treatment or had ER positive carcinomas and were therefore likely to have hormonally responsive disease. Although the inclusion of patients with a low probability of response to zindoxifene in the study reduced the likelihood of demonstrating activity of the compound, the complete absence of responders makes it unlikely that zindoxifene has useful activity in the treatment of breast cancer. On completion of zindoxifene treatment, 16 patients were considered suitable for further endocrine therapy. It is significant that five of these patients responded to treatment, including three of the six who were given tamoxifen.

Circulating zindoxifene metabolites have been detected at all dose levels used. The measured rise in SHBG levels at the higher doses of zindoxifene implies that sufficient drug is absorbed to have a biological effect. The significance of the rapid metabolism of zindoxifene is not understood. Although similar metabolism occurs in the rat, low levels of circulating free drug have been detected in that species, in contrast to humans (Birnbock *et al.*, 1987). It is possible that the difference in metabolism of the active free drug, D15414, between human and rat, although small, is sufficient to explain the different clinical results in the two species.

Serum SHBG levels showed a strong tendency to rise at the higher doses of zindoxifene used. A similar rise in SHBG levels with an associated fall in gonadotrophin levels in patients treated with tamoxifen has been attributed to the partial oestrogen agonist activity of tamoxifen (Sakai *et al.*,

1978; Willis *et al.*, 1977; Coombes *et al.*, 1982; Dowsett *et al.*, 1984). We have not observed a fall in gonadotrophin levels in zindoxifene treated patients. Since the compound is given orally, its effect on hepatic function (including SHBG synthesis) would be expected to be greater than its systemic activity, especially if this is reduced as a result of hepatic conjugation. This may explain the lack of a significant change in gonadotrophin levels despite the oestrogenic effect on SHBG levels. It is possible that zindoxifene has a predominantly oestrogenic rather than anti-oestrogenic action in humans, while at the same time failing to possess sufficient oestrogenic potency to induce tumour regression at the doses used. Recently reported *in vitro* studies using human cell lines support this interpretation (Robinson *et al.*, 1988). The lack of effect of zindoxifene on gonadotrophin levels (in contrast to that of tamoxifen) suggests that the *in vivo* oestrogenic activity of the compound is low, possibly as a result of hepatic metabolism. It would be interesting to compare the dose related oestrogenic effects of tamoxifen and zindoxifene. Unfortunately we have insufficient data to allow that in the current study.

Due to the phase I nature of the study, it is difficult to be certain that patients were treated with an optimal dose of zindoxifene, despite the variable dose escalation that we used. Nausea, which affected 50% of patients treated with 80 mg daily, precluded dose escalation above that level. Experimental studies in the rat, comparing zindoxifene to tamoxifen, suggest that both drugs should be equipotent at equimolar doses (Hilgard *et al.*, 1988). Doses as low as 20 mg daily could be expected to be active. Because of the dose escalations used in the current study, a minority of patients were treated for brief periods only with these low doses. It remains possible that we may have failed to demonstrate a therapeutic action of zindoxifene at the lower end of the dose range, although loss of therapeutic activity with increasing dose has not been described in the case of tamoxifen.

Tamoxifen is known to have a number of actions, such as inhibition of protein kinase-C and calmodulin, promotion of phospho-inositide hydrolysis and interaction with some classes of histamine, dopamine and acetylcholine receptors at the cell membrane, which are not mediated by interaction with the ER (Weiss *et al.*, 1988). Some of the non-genomic actions of tamoxifen may contribute to its activity in breast cancer and it may be that zindoxifene, an indole derivative which is structurally dissimilar to other anti-oestrogens, lacks such actions, accounting for some of its poor activity in breast cancer.

### References

BIRNBOCK, H., RINGSHANDL, R. & VON ANGERER, E. (1987). Chromatographic analysis of the new antiestrogen zindoxifene and its metabolites in biological material. *J. Chromatogr.*, **414**, 235.

COOMBES, R.C., POWLES, T.J., REES, L.H. & 6 others (1982). Tamoxifen, aminoglutethimide and danazol: effects of therapy on hormones in postmenopausal patients with breast cancer. *Br. J. Cancer*, **46**, 30.

DOWSETT, M., HARRIS, A.L., SMITH, I.E. & JEFFCOATE, S.L. (1984). Endocrine and clinical consequences of combination tamoxifen – aminoglutethimide in postmenopausal breast cancer. *Br. J. Cancer*, **50**, 357.

DOWSETT, M., ATTREE, S.L., VIRDEE, S.S. & JEFFCOATE, S.L. (1985). Oestrogen-related changes in sex hormone binding globulin levels during normal and gonadotrophin-stimulated menstrual cycles. *Clin. Endocrinol.*, **23**, 303.

DOWSETT, M., GOSS, P.E., POWLES, T.J., BRODIE, A.M.H., JEFFCOATE, S.L. & COOMBES, R.C. (1987). Use of the aromatase inhibitor 4-hydroxyandrostenedione in postmenopausal breast cancer: optimisation of therapeutic dose and route. *Cancer Res.*, **47**, 1957.

FERGUSON, K., HAYES, M. & JEFFCOATE, S.L. (1982). A standardised multicentre procedure for plasma gonadotrophin radioimmunoassay. *Ann. Clin. Biochem.*, **19**, 358.

HARTMANN, R.W. (1983). Tumor growth-stimulating and inhibiting effects of antiestrogens on the DMBA-induced mammary carcinoma of the ovariectomized, diethylstilbestrol-treated SD rat. A study on the mechanism of action of antiestrogens. *Eur. J. Cancer Clin. Oncol.*, **7**, 959.

HAYWARD, J.L., CARBONE, P.P., HEUSON, J.-C., KUMAOKA, S., SEGALOFF, A. & RUBENS, R.D. (1977). Assessment of response to therapy in advanced breast cancer. *Cancer*, **39**, 1289.

HILGARD, P., STEKAR, J., WUTTKE, W., ELLIGER, M. & HOLTEI, W. (1988). Comparative pharmacology of zindoxifene and tamoxifen. In *Progress in Cancer Research and Therapy, Vol. 35: Hormones and Cancer 3*, Bresciani, F., King, R.J.B., Lippman, M.E. & Raynaud, J.-P. (eds.) p. 369. Raven Press: New York.

JORDAN, V.C. (1984). Biochemical pharmacology of antiestrogen action. *Pharmacol. Rev.*, **36**, 245.

MCGUIRE, W.L. (1978). Steroid receptors in human breast cancer. *Cancer Res.*, **38**, 4289.

MOURISDEN, H., PALSHOF, T., PATTERSON, J. & BATTERSBY, L. (1978). Tamoxifen in advanced breast cancer. *Cancer Treat. Rev.*, **5**, 131.

ROBINSON, S.P., KOCH, R. & JORDAN, V.C. (1988). *In vitro* estrogenic actions in rat and human cells of hydroxylated derivatives of D16726 (zindoxifene), an agent with known antimammary cancer activity *in vivo*. *Cancer Res.*, **48**, 784.

SAKAI, F., CHEIX, F., CLAVEL, M. & 4 others (1978). Increases in steroid binding globulins induced by tamoxifen in patients with carcinoma of the breast. *J. Endocrinol.*, **76**, 219.

TAYLOR, R.E., POWLES, T.J., HUMPHREYS, J. & 5 others (1982). Effects of endocrine therapy on steroid-receptor content of breast cancer. *Br. J. Cancer*, **45**, 80.

VON ANGERER, E. (1984). Development of new drugs for endocrine tumour chemotherapy. *Cancer Treat. Rev.*, **11**, suppl. A, 147.

VON ANGERER, E., PREKAJAC, J. & BERGER, M. (1985). The inhibitory effect of 5-acetoxy-2-(4-acetoxyphenyl)-1-ethyl-3-methylindole (D16726) on estrogen-dependent mammary tumors. *Eur. J. Cancer Clin. Oncol.*, **21**, 531.

WEISS, D.J. & GURPIDE, E. (1988). Non-genomic effects of estrogens and antiestrogens. *J. Steroid Biochem.*, **31**, 671.

WILLIS, K.J., LONDON, D.R., WARD, H.W.C., BUTT, W.R., LYNCH, S.S. & RUDD, B.T. (1977). Recurrent breast cancer treated with the antiestrogen tamoxifen: correlation between hormonal changes and clinical course. *Br. Med. J.*, **i**, 425.